# Correcting Judgment Correctives in National Security Intelligence

**DOI:** 10.3389/fpsyg.2018.02640

**Published:** 2018-12-21

**Authors:** David R. Mandel, Philip E. Tetlock

**Affiliations:** ^1^Intelligence Group, Intelligence, Influence and Collaboration Section, Defence Research and Development Canada, Toronto, ON, Canada; ^2^Wharton School, University of Pennsylvania, Philadelphia, PA, United States

**Keywords:** judgment and decision making, intelligence analysis, debiasing, error management, corrective action, organizational policies

## Abstract

Intelligence analysts, like other professionals, form norms that define standards of tradecraft excellence. These norms, however, have evolved in an idiosyncratic manner that reflects the influence of prominent insiders who had keen psychological insights but little appreciation for how to translate those insights into testable hypotheses. The net result is that the prevailing tradecraft norms of best practice are only loosely grounded in the science of judgment and decision-making. The “common sense” of prestigious opinion leaders inside the intelligence community has pre-empted systematic validity testing of the training techniques and judgment aids endorsed by those opinion leaders. Drawing on the scientific literature, we advance hypotheses about how current best practices could well be reducing rather than increasing the quality of analytic products. One set of hypotheses pertain to the failure of tradecraft training to recognize the most basic threat to accuracy: measurement error in the interpretation of the same data and in the communication of interpretations. Another set of hypotheses focuses on the insensitivity of tradecraft training to the risk that issuing broad-brush, one-directional warnings against bias (e.g., over-confidence) will be less likely to encourage self-critical, deliberative cognition than simple response-threshold shifting that yields the mirror-image bias (e.g., under-confidence). Given the magnitude of the consequences of better and worse intelligence analysis flowing to policy-makers, we see a compelling case for greater funding of efforts to test what actually works.

## Introduction

Intelligence organizations in government play a vital role in informing the upper echelons of policymaking, the leaders of nations and their staff who are vested with the responsibility of protecting national security and promoting national interests. Within a given nation, the collective of intelligence organizations – euphemistically known as the intelligence community or, simply, the IC – therefore has an epistemic mandate to deliver timely, relevant, and accurate information to decision makers who operate under time and accountability pressures, the fog of uncertainty, and with foreknowledge that their decisions may alter the course of history.

How then has the IC sought to guarantee for policymakers and the public that they are doing their best to meet their epistemic mandate, given that the vast majority of substantive intelligence relies on human judgments made under conditions of deep uncertainty (Kent, 1964)? Do the IC’s tactics to ensure judgment quality rest on sound strategy properly informed by key concepts, methods and findings from judgment and decision science, the field that speaks directly to the challenges the IC faces? To the latter question, we believe the answer is – No. Yet we also remain optimistic that the IC could substantially improve the quality of its judgments if it took appropriate steps to correct its current corrective strategy – steps that we lay out as a set of IC policy prescriptions.

## The IC’s Current Corrective Approach

The IC is well aware both that its primary analytic product is judgment to support decision-making and that human judgment is prone to bias and error. Sherman Kent, an historian recruited to the fledgling IC during World War II and now widely regarded as the founder of modern intelligence analysis, was keenly concerned about the threats that confirmation bias and groupthink posed to epistemic integrity (Scoblic, 2018). Richards Heuer Jr. went further, documenting in *Psychology of Intelligence Analysis* (Heuer, 1999) how cognitive biases, much of which were revealed in the heuristics-and-biases research program inspired by Kahneman et al. (1982), could skew intelligence judgments and raise the risk of intelligence failure.

Heuer and others improvised simple, back-of-the-napkin, judgment-support methods that analysts could self-apply to debias their judgments and consequently improve their accuracy. The methods, which came to be known as structured analytic techniques or SATs, have proliferated (see Heuer and Pherson, 2014) and continue to represent the IC’s main tactical approach to combatting judgment error. In the United States, the Intelligence Reform and Terrorism Prevention Act of 2004 mandated use of SATs and many of them are presented to analysts in intelligence training as methods for coping with their unavoidable “mindsets and biases” (Marchio, 2014; Coulthart, 2017; Chang et al., 2018). More recently, Intelligence Community Directive 203 on analytic standards, promulgated by the Office of the Director of National Intelligence (ODNI), states that analysts “must employ reasoning techniques and practical mechanisms that reveal and mitigate bias” (Office of Director of National Intelligence [ODNI], 2015, p. 2), by which they mean SATs. Variants of this approach have spread to many other nations (e.g., Butler, 2004), an excellent example of a phenomenon that sociologists dub “institutional isomorphism.” The SAT paradigm has spread not because there is evidence it works, but because influential professionals in the most powerful organization have endorsed it and no one wants to fall behind prevailing norms of best practices. In these environments, pressures for interoperability can easily trump systematic searches for optimal design, resulting in suboptimal cross-organizational learning.

## Critique of the Current Approach

The IC’s current approach to judgment correctives is flawed for several reasons. We focus here on those that apply to the IC’s general approach to judgment correction and do not descend into the weeds to critique individual SATs. Given space constraints, we condense our arguments into two areas of critique: core organizational limitations and core conceptual limitations. These areas are related, and have a common denominator in the IC’s slow uptake from judgment and decision science, which followed from its commitment to an incidental approach, or lack of interest in pursuing a sustained, programmatic, and scientific approach to tradecraft innovation. We briefly address that common denominator before turning to the two areas of critique.

### The Incidental Approach to IC Innovation

The IC’s current approach to judgment correctives emerged from the attention of a handful of diligent analysts to specific problems they encountered in the practice of intelligence from the 1940s to 1980s. For instance, Kent’s stubborn preoccupation with improving the fidelity of communications of uncertainty estimates was affected by his direct experience with a policy-maker who was unsure of the meaning of the expression, “serious possibility,” that appeared in a 1951 National Intelligence Estimate on the probability of a Soviet invasion of Yugoslavia that year (Kent, 1964). When Kent asked his colleagues on the Board of National Estimates what they thought the term meant, he got answers ranging from 1:4 to 4:1 odds, which Kent described as jolting. Similarly, Heuer’s interest in intelligence tradecraft – and “alternative analysis,” in particular – was sparked by his involvement in the case of Soviet KGB defector Yuri Nosenko and his conclusion that the United States IC made inadequate effort to consider alternative explanations for a string of suspicious events that seemed to support the conclusion that Nosenko was a KGB disinformation agent (Heuer, 1987).

These tradecraft mavericks deserve credit for their trailblazing efforts to improve the practice of intelligence analysis. However, their examples also lay bare the adverse consequences of an *ad hoc*, character-driven approach to developing tradecraft. Critically, none of these tradecraft developers had advanced expertise in judgment and decision science. For example, although Heuer was well read in literature on higher-order cognition, he did not pursue it at a professional or even post-graduate level, and he was not trained in research methods and statistical analysis. It is therefore unsurprising that he did not subject his methods – notably the Analysis of Competing Hypotheses (ACH) technique – to experimental tests of whether they actually improved judgment in measurable ways.

### Organizational Limitations

Testing hypotheses is fundamental to both basic and applied sciences. Even our best ideas need to be put to rigorous empirical tests because most good ideas still fail. Mandel (in press) recently argued that the IC’s approach to tradecraft development follows what he called the *goodness heuristic*. Using this heuristic, if, upon mental inspection, an idea such as an imagined SAT for debiasing judgment seems good, then one should act on it as if it were in fact good because it probably is good. The goodness heuristic, which rests on a very likely excessively optimistic prior probability for ideational success, therefore takes Kahneman’s (2011) WYSIATI (what-you-see-is-all-there-is) principle to the next level by elucidating its implications for action by individuals and organizations.

Yet, as any seasoned scientist knows, not only do good ideas need to be rigorously tested, they need to be tested using multi-task and multi-benchmark methods (e.g., Mellers et al., 2017). There also should ideally be a diverse pool of ideas being tested by independent clusters of researchers, and among those clusters there must be a healthy sense of competition in epistemic tournaments, whether organized or *ad hoc* (e.g., Tetlock et al., 2017). This is vital because scientists, as theorists, can become prisoners of their preconceptions all too easily (Tetlock and Henik, 2005). Moreover, scientists, like all individuals, pursue goals other than purely epistemic ones (Mandel and Tetlock, 2016). It is vital, therefore, that scientists’ ideas and key findings be subject to peer scrutiny.

Those who shaped the IC’s current approach to judgment correctives varied in their commitment to testing ideas scientifically. Heuer, who had the greatest direct impact on the SAT approach to judgment correctives, questioned the value of science in adjudicating on the merits of proposed corrective methods. In an August 15, 2010 response to suggestions posted on an online discussion of the International Association for Intelligence Education that his ACH technique be empirically tested, Heuer wrote:

Can’t we have confidence in making a common sense judgment that going through the process of assessing the inconsistency of evidence will generally improve the quality of analysis? Similarly, can’t we have confidence in making a common sense judgment that starting the analysis with a set of hypotheses will, on average, lead to better analysis than starting by looking at the pros and cons for a single hypothesis? Do we really need an empirical analysis of these two points? Is it really feasible to do a high quality empirical analysis of the effectiveness of these two points?^1^

He also expressed reservations about the feasibility of experiments to test methods such as ACH, concluding, “If the empirical testing of my two claims about the value of ACH doesn’t replicate exactly how ACH is (or should be) used in the Intel Community, I would be inclined to ignore it and stick with my common sense judgment.”

It is ironic that one of the IC’s foremost tradecraft contributors, who stressed the importance of combatting confirmation bias, would take this stand. Yet the inconsistency should not shock us. The double standard – intuition is fine for me, but not for you – is simply more anecdotal evidence of the well-documented *bias blind spot*, the tendency to perceive biases in others’ thinking and judgments more easily than in one’s own (Pronin et al., 2002).

We do not blame Heuer and others for exhibiting what most of us exhibit to varying degrees, but his stance highlights a consequence of the IC’s decision over much of its history to invest very little in improving judgment quality through science, while investing heavily in collections technology. Over the last decade, the United States IC has changed this approach and now funds the Intelligence Advanced Research Projects Activity (IARPA), which is programmatic, engaging large numbers of scientists from industry and academia, and which has led to important scientific advances that hold promise for improving intelligence products. Whether these advances can be effectively integrated into the analytic training and workflows of intelligence organizations remains to be seen.

### Conceptual Limitations

The IC’s traditional approach to analytic tradecraft has also fostered conceptual setbacks. While a heavy emphasis is placed on the mitigation of cognitive biases, virtually no attention is given to the problem of imprecision and unreliability caused by “noisy” unsystematic error (Chang et al., 2018). Moreover, cognitive biases are conceptualized as unipolar phenomena needing to be reduced rather than as bipolar phenomena in which bias reduction strategies would require knowing where one was starting from, both in terms of direction and magnitude. Consequently, undue faith has been placed in assumptions regarding what types of biases needed to be corrected. For instance, whereas overconfidence is seen as problematic and attention is drawn to it in analytic training, the polar-opposite bias, underconfidence, is virtually ignored. However, recent studies show evidence of underconfidence in strategic intelligence forecasts (Mandel and Barnes, 2014, 2018) and in intelligence analysts’ probability judgments in experimental tasks (Mandel, 2015).

When we look at the research literature on how people cope with accountability demands (Lerner and Tetlock, 1999), we worry that the IC’s indiscriminate injunctions to beware of overconfidence will mainly yield indiscriminate response-threshold shifts – and the mirror-image bias of underconfidence. The net effect will be to further water down the informativeness of intelligence assessments for decision makers with excessive uncertainty. Similarly, the main effect of broad-brush warnings about confirmation bias might well be to induce endless second-guessing, to the point of analysis paralysis. Ultimately, the unipolar view of cognitive bias has allowed the IC to conveniently skirt value-laden, vexing questions about how bias-reduction tradeoffs should be resolved.

The IC’s error-neglect blind spot is equally troubling. Not only has the IC not taken proactive measures to minimize noise in intelligence judgments, noise neglect signals that the IC has not carefully considered how the very techniques they promote to minimize bias might amplify noise (Chang et al., 2018). Yet the weakly defined multistep processes that most SATs represent are no less than covert greenhouses for noise production. While giving the appearance of a standardized judgment-support process, SATs actually leave a long list of implementation decisions to analysts. How much agreement is there among analysts on such decisions? How reliably do the same analysts make these decisions over time? The few extant studies do not inspire optimism. For example, analysts asked to judge the probability of information accuracy on the basis of Admiralty-code ratings of source reliability (i.e., A–F) and information credibility (i.e., 1–6) were unreliable when the two ratings were incongruent in ordinal value, and inter-analyst agreement plummeted as scale incongruence increased (Mandel, 2018, Annex D).

In comparison to the Admiralty code, SATs like ACH create vast opportunities for inconsistency to flourish. To take just one example, consider the engine of ACH, which involves listing evidence in rows, hypotheses in columns, and then assessing the degree of consistency in each cell of the matrix. The meaning of consistency is left up to the analyst to interpret. One might treat it as the probability of the evidence given the hypothesis, while another might treat it as the inverse of that probability. Another still might assess whether the hypothesis necessarily follows from the evidence or vice versa, while yet another might run the test but with plausibility substituting for necessity. Perhaps the most common approach is to judge the representativeness of one to the other. In that case, and not without a touch of irony, ACH would be promoting the use of the representativeness heuristic under the guise of a debiasing strategy.

## Correcting the IC’s Current Corrective Approach

Both the organizational and conceptual limitations of the IC’s approach to judgment correctives, in particular, and analytic tradecraft, in general, stem from its *ad-hoc*, unscientific and character-driven nature. For the IC to develop effective correctives, it should abandon the complacent strategy of waiting for the next Kent or Heuer to spontaneously arise. The IC needs a diverse infusion of ideas from scientists outside the IC. It needs those scientists not only to put forward their best ideas, but also to test them in rigorous experiments or experimental tournaments. The IC should take the most promising results and work with scientific teams to transition the ideas into analytic processes. Those teams should also work with their IC counterparts to devise rigorous ways of trialing those processes, and the results of those trials should be taken seriously. What might work in an IARPA tournament, might not work so well in practice. If not, then reasons for variance in efficacy should be examined. Is the original idea doomed to transition failure, or was the transition strategy flawed but correctable?

The IC also should abandon the assumption that analytic judgments made in the absence of SATs must be intuitive and flawed. They should further banish the corollary view that although a SAT might not be perfect, it’s better than nothing. The first assumption is certainly wrong and the second is probably wrong too. While intuitive processes enter into analysts’ judgments, surely so can deliberative thought. SATs foster the illusion that intuition is driven from the judgment process. In reality, it is likely transferred to the process of conducting the SAT exercise itself. The effects of such transfer can be far from banal. For instance, SATs might disrupt good deliberative reasoning about the substantive issues. They might bolster undeserved confidence in the accuracy and logical coherence of analysts’ judgments. And they might foster IC complacency through the belief that corrective measures are sound and sufficient. For example, Mandel et al. (2018) report that intelligence analysts who were trained in ACH and who were instructed to use ACH to solve a probabilistic hypothesis-testing task were significantly more susceptible to coherence-violating unpacking effects (Tversky and Koehler, 1994) than a control sample of analysts from the same cohort who were not trained in ACH and who were left to their own reasoning devices.

Finally, the IC should broaden its horizons and start thinking beyond the analyst. All SATs share a focus on supporting the analyst, whether individually or in teams. Yet no attention has been given to how intelligence organizations might improve the accuracy of assessments through a range of post-analytic means such as recalibrating probabilistic judgments to correct for observable biases and aggregating judgments to boost signal-to-noise ratios through error cancelation and performance-sniffing methods. Recalibrating forecasts to make them more extreme has been shown to improve calibration in IARPA’s “ACE” geopolitical forecasting tournament (Baron et al., 2014; Turner et al., 2014) and in actual strategic intelligence forecasts (Mandel and Barnes, 2014). Likewise, recalibration methods that “coherentize” probability judgments by forcing them to respect one or more axioms of probability calculus, such as additivity and unitarity, can improve accuracy (Karvetski et al., 2013). The IC could also leverage decades of research on the benefits of statistically aggregating probability estimates. Taking an unweighted arithmetic average of multiple estimates is a highly effective method of error cancelation (Clemen and Winkler, 1999). More sophisticated aggregation methods that exploit individual differences in coherence (Predd et al., 2008; Wang et al., 2011; Karvetski et al., 2013) or other measurable aspects of performance (Cooke and Goossens, 2008) also hold promise for the IC. Indeed, Mandel et al. (2018) found that analysts’ judgment accuracy was substantially improved by first coherentizing and then aggregating their judgments.

To accelerate the discovery process, the IC should also take steps to systematically monitor the accuracy of its products. This will reveal the types of corrective actions most needed, and it can also shed light on factors that predict judgment accuracy. The results may be counter-intuitive and impossible to predict from theory. For instance, contrary to intuitive expectation, topic-related expertise among cancer research experts did not predict better accuracy in forecasting the reproducibility of cancer trial results, but expertise defined in terms of publication impact (h-index) did (Benjamin et al., 2017). Likewise, Tetlock (2005) found that political experts working inside their self-described domain of competence were no more accurate than experts working outside their domain in a geopolitical forecasting tournament. Ferreting out the factors that could be used in performance-sniffing weighting methods will take time and research effort, but these and other post-analytic interventions could significantly boost the IC’s judgment accuracy in years to come. The IC only needs to reduce the probability of a trillion-dollar mistake by a tiny amount to justify multi-million-dollar research investments.

## Author Contributions

Both authors contributed to the thinking behind and writing of this article.

## Conflict of Interest Statement

The authors declare that the research was conducted in the absence of any commercial or financial relationships that could be construed as a potential conflict of interest.

## Footnotes

1 Heuer, R. J., Jr., August 15, 2010 email correspondence sent to the International Association for Intelligence Education.

## References

[B1] Baron J. Mellers B. A. Tetlock P. E. Stone E. Ungar L. H. (2014). Two reasons to make aggregated probability forecasts more extreme. *Decis. Anal.* 11 133–145. 10.1287/deca.2014.0293

[B2] Benjamin D. Mandel D. R. Kimmelman J. (2017). Can cancer researchers accurately judge whether preclinical reports will reproduce? *PLoS Biol.* 15:e2002212. 10.1371/journal.pbio.2002212 28662052 PMC5490935

[B3] Butler L. (2004). *Review of Intelligence on Weapons of Mass Destruction: Report of a Committee of Privy Councillors.* London: The Stationery Office.

[B4] Chang W. Berdini E. Mandel D. R. Tetlock P. E. (2018). Restructuring structured analytic techniques in intelligence. *Intell. Natl. Secur.* 33 337–356. 10.1080/02684527.2017.1400230

[B5] Clemen R. T. Winkler R. L. (1999). Combining probability distributions from experts in risk analysis. *Risk Anal.* 19 187–203. 10.1111/j.1539-6924.1999.tb00399.x 10859775

[B6] Cooke R. M. Goossens L. L. H. J. (2008). TU Delft expert judgment data base. *Reliabil. Eng. Syst. Saf.* 93 657–674. 10.1016/j.ress.2007.03.005

[B7] Coulthart S. J. (2017). An evidence-based evaluation of 12 core structured analytic techniques. *Int. J. Intell. CounterIntell.* 30 368–391. 10.1080/08850607.2016.1230706

[B8] Heuer R. J. Jr. (1987). Nosenko: five paths to judgment. *Stud. Intell.* 31 71–101.

[B9] Heuer R. J. Jr. (1999). *Psychology of Intelligence Analysis.* Washington, DC: Center for the Study of Intelligence.

[B10] Heuer R. J. Jr. Pherson R. H. (2014). *Structured Analytic Techniques for Intelligence Analysis.* Washington, DC: CQ Press.

[B11] Kahneman D. (2011). *Thinking, Fast and Slow.* New York, NY: Farrar, Straus and Giroux.

[B12] Kahneman D. Slovic P. Tversky A. (1982). *Judgment Under Uncertainty: Heuristics and Biases.* Cambridge: Cambridge University Press. 10.1017/CBO9780511809477 17835457

[B13] Karvetski C. W. Olson K. C. Mandel D. R. Twardy C. R. (2013). Probabilistic coherence weighting for optimizing expert forecasts. *Decis. Anal.* 10 305–326. 10.1287/deca.2013.0279

[B14] Kent S. (1964). “Words of estimative probability,” in *Sherman Kent and the Board of National Estimates: Collected Essays*, ed. Steury D. P. (Washington, DC: Center for the Study of Intelligence), 133–146.

[B15] Lerner J. S. Tetlock P. E. (1999). Accounting for the effects of accountability. *Psychol. Bull.* 125 255–275. 10.1037/0033-2909.125.2.255 10087938

[B16] Mandel D. R. (2015). Instruction in information structuring improves Bayesian judgment in intelligence analysts. *Front. Psychol.* 6:387. 10.3389/fpsyg.2015.00387 25904882 PMC4389401

[B17] Mandel D. R. (2018). “Annex g: report on sas-114 experiment on analysis of competing hypotheses,” *in Proceedings of the SAS-114 Workshop on Communicating Uncertainty, Assessing Information Quality and Risk, and Using Structured Techniques in Intelligence Analysis*, ed. Mandel D. R. (Brussels: NATO STO), 10.14339/STO-MP-SAS-114

[B18] Mandel D. R. (in press). “Can decision science improve intelligence analysis?,” in *Correcting Judgment Correctives in Intelligence: A Reader*, eds Coulthart S. Landon-Murray M. Van Puyvelde D. (Washington, DC: Georgetown University Press).

[B19] Mandel D. R. Barnes A. (2014). Accuracy of forecasts in strategic intelligence. *Proc. Natl. Acad. Sci. U.S.A.* 111 10984–10989. 10.1073/pnas.1406138111 25024176 PMC4121776

[B20] Mandel D. R. Barnes A. (2018). Geopolitical forecasting skill in strategic intelligence. *J. Behav. Decis. Mak.* 31 127–137. 10.1002/bdm.2055

[B21] Mandel D. R. Karvetski C. W. Dhami M. K. (2018). Boosting intelligence analysts’ judgment accuracy: what works, what fails? *Judgm. Decis. Mak.* 13 607–621.

[B22] Mandel D. R. Tetlock P. E. (2016). Debunking the myth of value-neutral virginity: toward truth in scientific advertising. *Front. Psychol.* 7:451. 10.3389/fpsyg.2016.00451 27064318 PMC4811882

[B23] Marchio J. (2014). Analytic tradecraft and the intelligence community: enduring value, intermittent emphasis. *Intell. Natl. Secur.* 29 159–183. 10.1080/02684527.2012.746415

[B24] Mellers B. A. Baker J. D. Chen E. Mandel D. R. Tetlock P. E. (2017). How generalizable is good judgment? A multi-task, multi-benchmark study. *Judgm. Decis. Mak.* 12 369–381.

[B25] Office of Director of National Intelligence [ODNI] (2015). *Intelligence Community Directive 203: Analytic Standards.* Washington, DC: Office of Director of National Intelligence.

[B26] Predd J. B. Osherson D. N. Kulkarni S. R. Poor H. V. (2008). Aggregating probabilistic forecasts from incoherent and abstaining experts. *Decis. Anal.* 5 177–189. 10.1287/deca.1080.0119

[B27] Pronin E. Lin D. Y. Ross L. (2002). The bias blind spot: perceptions of bias in self versus others. *Pers. Soc. Psychol. Bull.* 28 369–381. 10.1177/0146167202286008

[B28] Scoblic J. P. (2018). *Beacon and Warning: Sherman Kent, Scientific Hubris, and the Cia’s Office of National Estimates. Texas National Security Review.* Available at: https://tnsr.org/2018/08/beacon-and-warning-sherman-kent-scientific-hubris-and-the-cias-office-of-national-estimates/.

[B29] Tetlock P. E. (2005). *Expert Political Judgment: How Good Is It? How Can We Know?.* Princeton, NJ: Princeton University Press.

[B30] Tetlock P. E. Henik E. (2005). “Theory- versus imagination-driven thinking about historical counterfactuals: are we prisoners of our preconceptions?,” in *The Psychology of Counterfactual Thinking*, eds Mandel D. R. Hilton D. J. Catellani P. (New York, NY: Routledge).

[B31] Tetlock P. E. Mellers B. A. Scoblic J. P. (2017). Bringing probability judgments into policy debates via forecasting tournaments. *Science* 355 481–483. 10.1126/science.aal3147 28154049

[B32] Turner B. M. Steyvers M. Merkle E. C. Budescu D. V. Wallsten T. S. (2014). Forecast aggregation via recalibration. *Mach. Learn.* 95 261–289. 10.1007/s10994-013-5401-4

[B33] Tversky A. Koehler D. J. (1994). Support theory: a nonextensional representation of subjective probability. *Psychol. Rev.* 101 547–567. 10.1037/0033-295X.101.4.547

[B34] Wang G. Kulkarni S. R. Poor H. V. Osherson D. N. (2011). Aggregating large sets of probabilistic forecasts by weighted coherent adjustment. *Decis. Anal.* 8 128–144. 10.1287/deca.1110.0206

